# A mouse model of peripheral nerve injury induced by Japanese encephalitis virus

**DOI:** 10.1371/journal.pntd.0010961

**Published:** 2022-11-28

**Authors:** Xiaoli Wang, Guowei Wang, Huan Yang, Shihong Fu, Ying He, Fan Li, Huanyu Wang, Zhenhai Wang

**Affiliations:** 1 The NO.1 People’s Hospital of Shizuishan, Shizuishan, China; 2 Ningxia Medical University, Yinchuan, China; 3 General Hospital of Ningxia Medical University, Yinchuan, China; 4 Department of Arbovirus, NHC Key Laboratory of Biosafety, National Institute for Viral Disease Control and Prevention, Chinese Center for Disease Control and Prevention, Beijing, China; 5 State Key Laboratory for Infectious Disease Prevention and Control, Chinese Center for Disease Control and Prevention, Beijing, China; 6 Neurology Center, General Hospital of Ningxia Medical University, Yinchuan, China; 7 Diagnosis and Treatment Engineering Technology Research Center of Nervous System Diseases of Ningxia Hui Autonomous Region, Yinchuan, China; NIAID Integrated Research Facility, UNITED STATES

## Abstract

Japanese encephalitis virus (JEV) is the most important cause of acute encephalitis in Eastern/Southern Asia. Infection with this virus also induces peripheral nerve injury. However, the disease pathogenesis is still not completely understood. Reliable animal models are needed to investigate the molecular pathogenesis of this condition. We studied the effect of Japanese encephalitis virus infection in C57BL/6 mice after a subcutaneous challenge. Limb paralysis was determined in mice using behavioral tests, including a viral paralysis scale and the hanging wire test, as well as by changes in body weight. Nerve conduction velocity and electromyography testing indicated the presence of demyelinating neuropathy of the sciatic nerve. Pathological changes in neural tissues were examined by immunofluorescence and transmission electron microscopy, which confirmed that the predominant pathologic change was demyelination. Although Western blots confirmed the presence of the virus in neural tissue, additional studies demonstrated that an immune-induced inflammatory response resulted in severe never injury. Immunofluorescence confirmed the presence of Japanese encephalitis virus in the brains of infected mice, and an inflammatory reaction was observed with hematoxylin-eosin staining as well. However, these observations were inconsistent at the time of paralysis onset. In summary, our results demonstrated that Japanese encephalitis virus infection could cause inflammatory demyelination of the peripheral nervous system in C57BL/6 mice.

## Author summary

Since first being isolated in 1935, Japanese encephalitis virus (JEV) has primarily been associated with encephalitis and has caused repeated outbreaks in East and Southeast Asia. In 2018, a Japanese encephalitis (JE) outbreak emerged in Ningxia province of China. Unlike previous outbreaks, in the 2019 outbreak, numerous patients presented with Guillain-Barre syndrome (GBS). Although the acute encephalitis syndrome caused by JEV has been studied extensively, GBS induced by JEV is an uncommon manifestation. No approved effective antiviral therapy directed against JEV is currently available. Therefore, we successfully constructed a mouse model of peripheral nerve injury (PNI) induced by JEV that can be used to study the pathogenic mechanism of the virus and develop new therapeutic targets.

## Introduction

Japanese encephalitis virus (JEV) is a mosquito-borne, single-stranded positive-polarity RNA virus. Japanese encephalitis (JE), caused by JEV infection, is the major cause of viral encephalitis in East and Southeast Asia [1]. Approximately 68,000 people are infected with JEV each year [2], which is a considerable threat to public health in Asia.

JE exhibits a broad spectrum of clinical manifestations ranging from non-specific acute febrile illness to a life-threatening acute encephalitis syndrome. In addition, some patients experience various signs of focal neurological deficits, such as Parkinson’s disease [3], polio-like flaccid paralysis [4–6], and cranial nerve damage [7]. Five genotypes of JEV have been identified and described as G I through G V [8,9]. In the past, the primary virus genotype that caused JE was G III. Recently, G I has gradually replaced G III as the predominant pathogenic genotype [10–12]. Alterations in the predominant genotype might be the underlying cause of the observed changes in the clinical characteristics. As seen with the JE epidemic in Ningxia province of China in 2018 [13], some patients developed symptoms similar to Guillain-Barré syndrome (GBS), including flaccid limb paralysis and respiratory muscle paralysis. Additional studies confirmed that these patients were associated with JEV infection [14]. Sporadic clinical case reports have recently revealed an association between the two diseases [15]. However, to our knowledge, the mechanism of JEV-induced peripheral nerve injury (PNI) has not been investigated.

A member of the flaviviridae family, JEV has strong neurotropic and neuroinvasive characteristics, similar to Zika, Dengue, and West Nile viruses [16]. Zika virus has been associated with GBS [17,18], and PNI has been reported to be associated with Dengue virus [19,20] and West Nile virus [21,22]. Since JEV shares a similar genomic structure with other flaviviruss, it might also cause peripheral neuropathy and GBS.

Currently, there is no specific and effective therapy for JEV infections. Due to the diverse clinical manifestations induced by JEV infection and the current difficulties in treatment, finding a more effective approach to treatment has become urgent. JEV infection can be prevented through vaccination, and existing vaccines are based on the G III virus. However, the observation that G I and re-epidemic G V are gradually replacing G III has raised concerns about the effectiveness of the vaccine [23,24].

We know that in vivo experiments in animal disease models are essential for viral pathogenesis studies. Different animal models for JE have been established and widely used in biological research. However, an animal model of JEV-induced PNI has not been reported. Over the past decades, considerable research has been conducted on JE and PNI. However, it has yet to be reported how JEV induces PNI. Hence, it is urgent to establish efficient animal models to study the pathogenesis. To this end, we developed a successful JEV-induced PNI mouse model utilizing a range of validated experimental methods.

## Materials and methods

### Animals and ethics approval

Sixty-eight male SPF-grade C57BL/6 mice aged 4 to 6 weeks were purchased from Beijing Vital River Laboratory Animal Technology Co., Ltd., and housed in the Laboratory Animal Facility at the Chinese Center for Disease Prevention and Control (China CDC). The mice were raised at standard room temperature with a 12 h alternating light/dark cycle, and food and water were freely available. All animal experiments were approved by the Ethics Committee of the National Institute for Viral Disease Control and Prevention of the Chinese Center for Disease Prevention and Control (20201110064 and 20210429031) and carried out in strict accordance with the requirements of the ethics committee. All animal experiments were conducted under bio-safety level-2 (BSL-2).

### Virus

The NX1889 strain of JEV was used in this study, which was a strain from Ningxia, that was isolated from a patient’s cerebrospinal fluid (CSF). Following the protocol described in one of our previous reports [14], the virus was propagated using C6/36 cells and Vero BHK-21 cells.

### Animal behavior

All mice were randomly divided into two groups: a control group (n = 18) and a virus-exposed group (n = 50). The initial body weight of each mouse was determined. Mice exposed to the virus were injected subcutaneously with JEV (105 pfu, 50 μl per mouse). Mice in the control group received an equivalent volume of PBS. All mice were observed daily and assessed for glossiness of the hair, activity level, and the presence of limb paralysis [25,26]. The mouse weights were recorded daily, and each mouse was evaluated based on the virus paralysis scale (VPS) and the hanging wire test. The 15-day period was separated into four phases: 1 day post injection (dpi)-4 dpi, 5 dpi-8 dpi, 9 dpi-12 dpi, and13 dpi-15 dpi. Some mice in each group were selected for follow-up experiments on days 4, 8, 12, and 15. Mice were euthanized when their VPS score was greater than 4 points, their body weight decreased by more than 1 g, and they appeared extremely weak. Based on these criteria, some mice were euthanized prior to the designated time for the conclusion of the experiment. They will be assigned to the phase where they reside on. The bilateral sciatic nerves and brains were obtained from each mouse.

### Viral paralysis scale (VPS)

A sensitive method was used to analyze the signs of tail and limb paralysis in mice [27]. Each mouse was placed on a table and allowed to walk freely for several minutes. The researchers scored the limb function according to the seven points of the scoring method detailed in Table 1. The score was based on four main aspects: tail position during walking, miss-step severity [28], weight-bearing, and joint movement. The severity of miss-steps was scored only in mice that could walk adequately, which was defined as a mouse that could travel three body lengths at a constant speed without turning [29].

**Table 1:** Viral paralysis scale [27].

Score	Description	Performance
0	Normal	Normal, weight-bearing, plantar steppinga with tail up during walking passesb
1	Onset of symptoms Tail position: Miss-step: Weight-bearing:	Weight-bearing, plantar stepping with mild rotation
		May be down or not fully up
		Foot rotated on take-off or landing
		Wobble is present indicating weakness
2	Mild paresis: Tail position: Miss-step: Weight-bearing: Joint movement:	Mild miss-steps (but able to bear weight)
		Down
		Mild, toe-curling/dragging on the ground, foot slightly skids medially or laterally
		A limp may be present indicating weakness
		May appear stiffer
3	Moderate paresis Miss-step: Weight-bearing: Joint movement:	Moderate miss-steps (but able to bear weight)
		Obvious foot curling/dragging on ground foot obviously skids medially or laterally
		Limb is obviously weak
		May appear stiffer
4	Severe paresis Miss-step: Weight-bearing: Joint movement:	Severe miss-steps (not bearing much weight)
		Limb mostly drags behind, medially or laterally
		Not much, but limb still used to aid forward motion
		Obviously decreased
5	Paralysis Miss-step: Weight bearing: Joint movement:	No weight-bearing steps, slight joint movement
		No stepping, limb only drags
		None
		Slight
6	Complete paralysis Joint movement:	No weight bearing step, no joint movement
		None

a Plantar stepping: Paw is placed flat on the ground during stepping. The foot does not curl or skid to one side, and the toes/feet do not curl or drag at any point

b Walking pass: The animal moves three body lengths at a consistent speed and without turning

### Hanging wire test

A transparent box was constructed with a height of 50 cm, a bottom, and an open top. The bottom was covered with a cotton cloth to prevent mice from being hurt when they fell. The top was covered with a 45 cm long and 30 cm wide wire mesh frame. The wire diameter of the grid was approximately 2 mm, and the area of the grid squares was 1 × 1 cm. The wire mesh frame just covered the top of the box. Each mouse was initially placed on the wire mesh frame and allowed to move freely. Then the frame was turned over (180°) and placed on the top of the box to observe the time the mouse stayed on the wire mesh frame [30,31]. The maximum time was 180 s, and remaining on the wire mesh frame for 180 s was considered normal. If the mouse fell during this period, the time to falling was recorded. The experiment was carried out three times, with an interval of 2 min between each experiment.The average time for the three repeated experiments was recorded [29]. After the trial, each mouse was returned to its cage.

### Electromyography

Electromyography (NDI-094 Shanghai Haishen), which is used to diagnose human peripheral neuropathy was utilized to detect the compound muscle action potential (CMAP) of the mouse sciatic nerve. Briefly, each mouse was anesthetized with an intraperitoneal injection of 10% chloral hydrate. After anesthesia, the mice were fixed on the surgical platform in the prone position, and the bilateral sciatic nerves were passively separated. A ground wire was fixed to the skin on the back of the mouse to eliminate possible interference. A recording bipolar needle electrode was inserted into the belly of the gastrocnemius muscle of one hindlimb. Then the stimulating electrode was placed sequentially in the proximal and sciatic nerve notch [32,33]. At the beginning of stimulation, the current was increased manually, starting from 0.1 mA and increasing progressively until a maximum amplitude was produced [34]. The observed waveform was recorded, and the distance between the two stimulation points of the sciatic nerve was measured and recorded. The amplitude, end latency, and conduction velocity of the sciatic nerves were obtained for each mouse tested [35].

### Immunofluorescence

Under anesthesia, one sciatic nerve and the mouse brains were removed and fixed in 4% paraformaldehyde. The fixed tissues were paraffin-embedded and sectioned. The sections were deparaffinized and placed in EDTA buffer (pH 8.0) for antigen retrieval. After cooling to room temperature, the sections were quenched with an autofluorescence. Non-specific binding was blocked by incubating the sections in bovine serum albumin (BSA) for 30 minutes. Then the sections were incubated with the corresponding primary antibodies overnight at 4°C. The primary antibodies included rabbit anti-MBP monoclonal antibody 1:5,000 (Abcam, UK, ab21801), rabbit anti-NF-H monoclonal antibody 1:5,000 (Abcam, UK, ab207176) [36], and mouse anti-Japanese encephalitis virus E glycoprotein monoclonal antibody 1:50 (Abcam, UK, ab41671). The next day, the primary antibody solution was removed, and secondary antibodies were added, including Alexa Fluor-488 goat anti-rabbit IgG (Servicebio, Wuhan, China, GB25303, green), Cy3 goat anti-rabbit IgG (Servicebio, Wuhan, China, GB21303, red), and Cy3 goat anti-mouse IgG (Servicebio, Wuhan, China, GB21301, red). Sections were incubated with fluorescent secondary antibodies for 50 min at room temperature. Then, nuclei were stained with DAPI for 10 min. Finally, the sections were coverslipped and sealed with an anti-fluorescence quenching sealing agent and observed under a fluorescence microscope (Nikon eclipse C1). Images were collected (Nikon da-u3), and Image J 180 software was utilized to measure the fluorescence levels. The average fluorescence intensity was calculated by dividing the total fluorescence intensity by the total fluorescent area of the cells.

### Transmission electron microscopy (TEM)

One sciatic nerve of each mouse was placed in an electron microscopy fixation solution at 4°C for 2 to 4 hours. After rinsing in 0.1 M phosphate buffer (PB) (pH 7.4), the tissues were postfixed with 1% osmic acid (OsO4) in 0.1 M PB for 2 h at room temperature. After dehydration and embedding, ultrathin sections were obtained and observed using a transmission electron microscope (HITACHI HT 7700) for image acquisition and analysis [37,38].

### Western blot analysis

Sciatic nerve tissues were ground in cold lysis buffer. After incubation on ice for 30 min, the proteins were recovered by centrifugation at 12,000g at 4°C for 10 min. Total proteins was quantified using the BCA method. Then proteins were denatured and separated by SDS-PAGE. A protein marker was added for molecular labeling. Tissues obtained from mice injected with PBS were used as the control. Proteins were separated by electrophoresis and transferred to PVDF membranes. The membranes were blocked in a solution of 5% non-fat milk, then incubated with primary antibodies, including rabbit anti-JEV-E polygonal antibody 1:10,000 (Hsinchu Taiwan, Taiwan, China, GTX125867), rabbit anti-MBP monoclonal antibody 1:1,000 (Abcam, UK, ab218011), rabbit anti-NF-H monoclonal antibody 1:1,000 (Abcam, UK, ab207176); rabbit anti-IL-1β monoclonal antibody 1:1,000 (Abcam, UK, ab283818), rabbit anti-IL-6 monoclonal antibody 1:1,000 (Abcam, UK, ab229381), and rabbit anti-TNF-α monoclonal antibody 1:1,000 (Abcam, UK, ab183218), at 4°C overnight. Subsequently, the membranes were incubated with corresponding secondary antibodies, goat anti-rabbit IgG 1:10,000 (Absin, China, abs20040ss). The gels were imaged using a Bio-Rad Gel Doc XR imager. The membranes were subjected to densitometry using Image J 180 software. GAPDH (1:10,000) served as an internal control, and the experiment was repeated three times.

### Hematoxylin and eosin (H&E) staining

After anesthesia, the mice were perfused with PBS followed by 4% paraformaldehyde. The fixed brains were removed from the skull, embedded in paraffin, using standard protocols, sectioned, and stained with hematoxylin and eosin (HE).

### Statistics

Data analyses were performed using GraphPad Prism 8.0.2 (GraphPad Prism software, La Jolla, CA, USA). All measured data were expressed as means ± standard deviation. Comparisons between the two groups were assessed using the Normality and Levene tests. If the data were determined to have a normal distribution, the t-test was used. If the data failed to pass the Levene test, the Welch t-test was used. The Wilcoxon rank-sum test was used for non-normal data. One-way analysis of variance (ANOVA) was used to compare multiple groups after applying the Normality and Levene tests. The Kruskal-Wallis H test was used on data that were not normally distributed. The Dunnett t-test was used for multiple comparisons between multiple groups. A value of P < 0.05 was considered statistically significant. The degree of difference was expressed as *p < 0.05, **p < 0.01, ***p < 0.001 and ****p < 0.0001.

## Results

### Mice inoculated with the virus exhibited signs of neurological disease

To determine the optimal volume of virus inoculation and the optimal times for disease assessment, we exposed mice to five different virus titers (102pfu, 103pfu, 104pfu, 105pfu, and 106pfu) and evaluated the inoculated mice for 20 days. Mice treated with PBS were used as controls (n = 5). Each titer was injected into five mice (n = 5). All mice inoculated with JEV were designated as the virus group (n = 25). We discovered that mice exhibited limb paralysis and general weakness in each virus titer group. The VPS scores for each titer are shown in S1A Fig. We found that the disease severity did not directly correlate with the viral titer. Furthermore, the mice that received 104pfu (p = 0.0345) and 105pfu (p = 0.0268) presented significant advantages when evaluated. From 1 dpi to 5 dpi, the VPS of the virus group exhibited scores of 0 to 2 points. From 6 dpi to 15 dpi, the scores were between 0 to 5 points, and after 15 dpi, the scores of the virus group were between 0 to 1 point (S1B Fig). These results indicated that some symptoms, such as limb paralysis and frailty, were more severe prior to 15 dpi. On the other hand, when the body weight changes were compared between the virus and control groups, we found that there was no significant difference between the two groups prior to 5 dpi. From 5 dpi to 15 dpi, the changes in body weight between the two groups became more pronounced, indicating that the body weight of the mice in the virus group increased more slowly than in the control group. After 15 dpi, the body weights of the mice in the virus group increased more rapidly (S2 Fig). These results suggested that the mice appeared to be recovering after 15dpi. Based on this information, the virus titer used in the experiment was 105pfu, the observation period was 15 days, and the time intervals were 4 dpi, 8 dpi, 12 dpi, and 15 dpi.

Behavioral changes in mice were easy to observe and could be used as an initial assessment to determine whether mice were ill. Based on daily observations of the general condition of the mice, we determined that the mice in the control group presented a shiny hair coat, were active, moved freely with a steady gait, and ran quickly. However, the mice in the virus-exposed group exhibited different degrees of rough hair coats, weakness, and reduced activity. Some exposed mice also exhibited a hunched back, body tremors, limping on one or both hind limbs, and a weakened forelimb grip [39].

**Fig 1:**
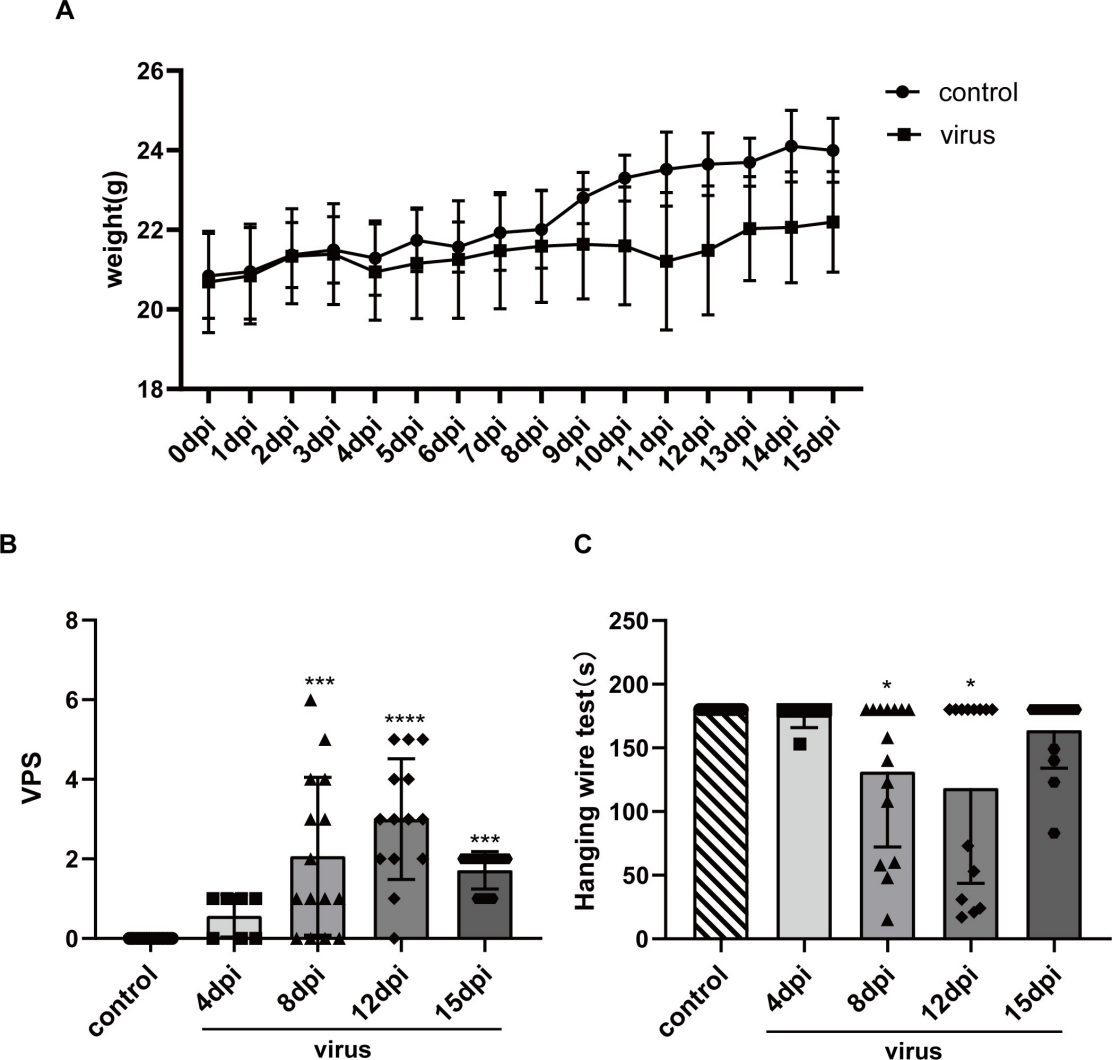
Behavioral changes of mice at different time. (A) Body weight change of the mice at different time; (B) VPS scores at different time. Kruskal-Wallis H test was used (****p< 0.0001); (C) Hanging wire test at different time. Kruskal-Wallis H test was applied for statistical analysis (**p = 0.005).

Body weight also was a good indicator of animal health [40]. The body weights of both mouse groups were recorded daily. Viral infection can lead to decreased appetite and weight loss in mice. The body weights were expressed as means ± standard deviation. We observed that mice in both groups gained weight. However, the weights of mice in the control group increased steadily, from 20.84g ± 1.07g at the beginning of the study to 22.58g ± 1.00g at the end of the investigation. The weights of the virus-exposed mice increased more slowly, with an initial weight of 20.69g ± 1.27g and a weight of 21.66g ± 0.38g at the end of the investigation, which was approximately 1g less than in the control group. Some mice exhibited significant weight loss following virus exposure. However, before 4 dpi, no significant difference in weight was observed between the two groups. While the mean body weight in the virus group was lower than the control group from 4 dpi to 8 dpi, both groups exhibited increased body weights. The mean body weight in the control group increased significantly after 8 dpi, but only a minimal increase or even a decrease in body weight was observed in the virus-exposed mice. The increase in body weight in the virus-exposed mice improved after 12dpi (Fig 1A).

In addition to observing the general condition of the mice, motor function was evaluated. Based on the viral paralysis scale (VPS), the mice in the control group (n = 18) did not exhibit any stiffness or paralysis of their limbs and received a score of 0 (normal). Virus-exposed mice exhibited progressively slower movements, tail dragging, stiff joints [40], lameness while walking, and an inability to perform normal plantar stepping. In some cases, the virus-exposed mice were unable to support their weight. The VPS ranged from 0 to 6 points. A score of ≤ 2 points indicated no apparent limb paralysis. A score of 3 to 6 points indicated the presence of limb paralysis. An increasing score indicated that the degree of paralysis increased in severity [29]. Compared to the control group at 4 dpi, the VPS of mice in the virus group was ≤ 1 point (n = 7, p > 0.9999). Three mice were scored as 0-points (3/7), and four received a score of 1-point (4/7). A lower tail position and a slight sway in their gait when the mice walked were observed. Starting at 8 dpi (n = 15, ***p = 0.0006), the number of mice with VPS between 3 to 6 points increased. Of the 15 mice that were scored, 4/15 were 0-points, 4/15 were 1-point, 1/15 were 2-points, 2/15 were 3-points, 1/15 were 5-points), and 1/15 were 6-points. Considerable limb paralysis was observed at this stage, including joint stiffness, significant weakness, limb dragging, reduced joint activity, and inability to bear weight. The VPS was between 0 to 5 points at 12 dpi (n = 14, ****p < 0.0001) and 1 to 2 points at 15 dpi (n = 14, ***p = 0.0002). The VPS assessments of 14 mice at 12 dpi were 1/14 as 0-points, 1/14 as 1-point, 3/14 as 2-points, 4/14 as 3-points, 2/14 as 4-points, and 3/14 as 5-points. At 15 dpi the scores were 4/14 as 1-point and 10/14 as 2-points (Fig 1B).

We conducted a hanging wire test, which was supplementary to the VPS assessment, to eliminate subjective bias. The motor function of the mouse limbs was assessed by recording the time each mouse was able to suspend its body on an inverted wire mesh. This test assessed the strength of their limbs, which was essential to keep them from falling. Mice in the control group exhibited a robust ability to grasp the wire mesh and were able to move continuously on the upside-down wire mesh frame without falling. The suspension time for the control mice reached even exceeded 180s. Numerous mice in the virus-exposed group fell more than once. The mice barely moved when hanging from the wire mesh and primarily stayed in one place. The lack of movement while suspended on the wire mesh might have helped maintain their limb strength to avoid falling. At 4 dpi (p > 0.9999), only one mouse fell once in the virus group and returned to normal when the experiment was repeated. On the other hand, starting at 8 dpi (*p = 0.0109), additional mice in the virus-exposed group fell one or more times. Some mice who fell on the first trial, occasionally stayed suspended on the wire mesh for 180s in the second or third trial of the day, but most mice fell all three times. Eight of 15 mice in the virus group spent less than 180s suspended on the wire mesh. At 12 dpi, the time the virus-exposed mice were able to suspend their bodies from the wire mesh also was significantly shorter (*p = 0.0283). Six of 14 mice fell and the time they remained suspended was less than 80s. At 15 dpi (p >0.9999), the amount of time the virus-exposed mice remained suspended on the wire mesh was longer than the average time observed at 12 dpi, and fewer mice fell (4/14) (Fig 1C).

The VPS and hanging wire test results indicated that, although the virus-exposed mice exhibited considerable limb paralysis (VPS ≥ 3), many mice did not exhibit a significantly shorter time during which they were able to suspend their bodies during the hanging wire test. This may be because mice also presented symptoms such as weakness and reduced activity, which increased the VPS score. However, these factors did not significantly influence the outcome of the hanging wire test. It was consistent that symptoms exhibited by the virus-exposed mice were most prominent from 8 dpi to 12 dpi, indicating that the virus-exposed mice presented considerable limb weakness during this period. After 12 dpi, mice with a score less than 3 points did not exhibit any discernable limb weakness, and the number of mice whose scores were between 1 and 2 points increased. Furthermore, the virus-exposed mice had sufficient limb strength to allow them to remain suspended from the wire mesh. Accordingly, there was no apparent reduction in the time the mice remained suspended during the hanging wire test.

### Abnormal CMAP of sciatic nerves in virus-exposed mice

**Fig 2:**
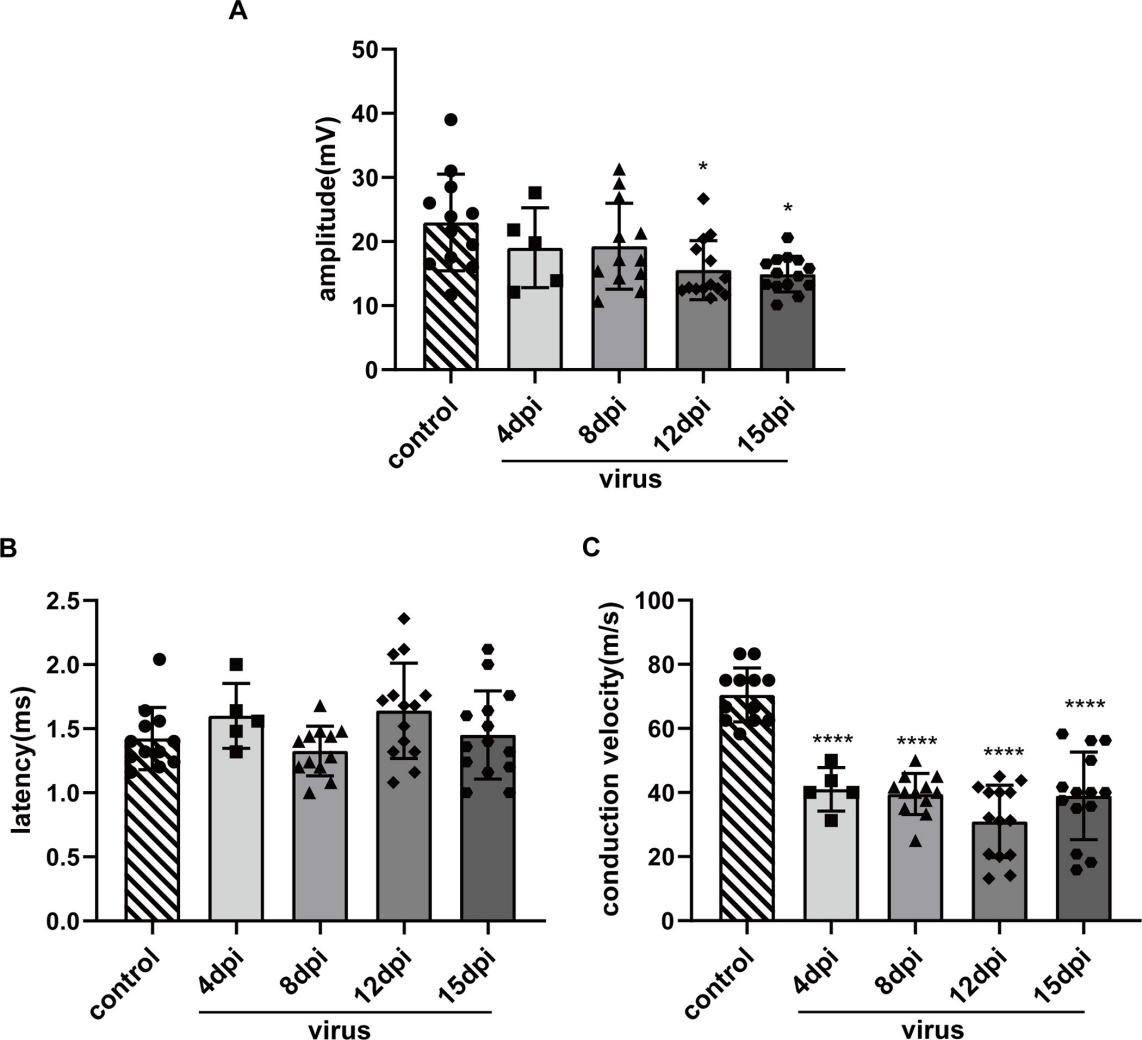
CMAP assessment of the sciatic nerve in mice. (A) Amplitude. Kruskal-Wallis H test was used (*P = 0.0173); (B) End latency. One-way ANOVA was used (P = 0.0903); (C) Conduction velocity. Statistical analysis by one-way ANOVA (****P < 0.0001).

Due to overlapping symptoms, it is often impossible to distinguish lesions of the central and peripheral nervous systems based only on behavioral observations. Electromyography (EMG) is an important assessment modality for the diagnosis of peripheral nerve diseases. It can identify various types of PNI at an early stage. We focused on the motor function of the sciatic nerve in this study and used EMGs to obtain the CMAP of mice at 4, 8, 12, and 15 dpi. The conduction function of motor nerves was determined based on analysis of the amplitude, end latency, and conduction velocity of the action potentials. We observed small differences in the results obtained with the sciatic nerves on both sides of the mice, but the differences did not reach statistical significance, which was similar to humans. These differences also were observed in the control mice, and the amplitude of the right side was typically slightly higher than the left side. Many factors can cause changes in waveforms [41]. Consequently, we only compared the results obtained on right side. The amplitude of the sciatic nerves in the virus group (n = 45) was lower than in the control group (n = 12), but the differences were relatively small from 4 dpi (n = 5, p >0.9999) to 8 dpi (n = 12, p >0.9999). The amplitude significantly decreased at 12 dpi (n = 14, *p = 0.0326) and 15 dpi (n = 14, *p = 0.0446) (Fig 2A). The latency of the sciatic nerves was not significantly prolonged (p = 0.1136). A prolonged latency was observed at some time points, but the differences were not statistically significant (Fig 2B). The conduction velocity of the sciatic nerves in the virus-exposed mice was significantly decreased at 4 dpi. The decrease was even more pronounced over time, but improved at 15 dpi (****p < 0.0001), although the differences between groups were not significant (Fig 2C). The conduction velocity, which reflected the function of the motor nerve myelin sheath, was significantly decreased in the early stage after virus exposure. In contrast, the amplitude, which represented axon function, decreased significantly only in the moderate-to-advanced stages. These results suggested that JEV infection primarily led to demyelination of the mouse sciatic nerves in the early period and mild axonal lesions appeared later.

### Reduction in fluorescence intensity of myelin sheaths and axons of the sciatic nerve occurred at different time points

**Fig 3:**
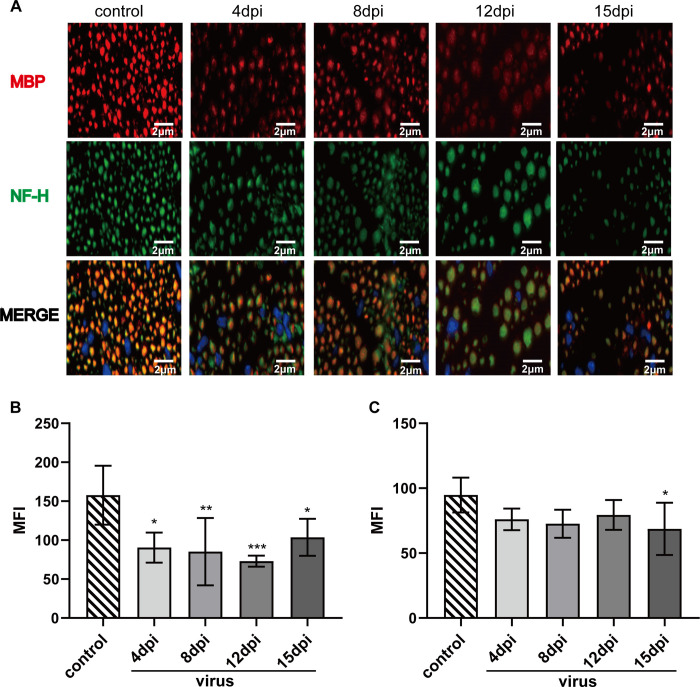
Immunofluorescence of mouse sciatic nerve at different time. One-way ANOVA was used. (A) The sciatic nerve tissue was double stained with MBP + NF-H and labeled with fluorescence. MBP was labeled in red and NF-H in green. Nuclei are stained DAPI (blue); (B) MFI of MBP (**P = 0.0015); (C) MFI of NF-H (P = 0.0666).

Myelin sheaths and axons were labeled with red and green fluorescent proteins, respectively, to understand the corresponding lesions, and the mean fluorescence intensities (MFI) were compared (Fig 3A). The MFI of MBP, a myelin-specific protein, was 157.75 ± 33.86, and NF-H, an axon-specific protein, was 94.78 ± 12.01 in the control group (n = 5). Compared with the control group, the MFI of MBP in the virus-exposed mice decreased significantly at 4 dpi (n = 3, *p = 0.0354). The expression continued to decline from 4 dpi to 12 dpi (8 dpi, n = 5, **p = 0.0066; 12 dpi, n = 6, ***p = 0.0009). However, the MBP expression was elevated at 15 dpi (n = 6, **p = 0.0433) compared with 12 dpi (Fig 3B). Although the NF-H expression levels appeared reduced from 4 dpi to 12 dpi (4 dpi, p = 0.3927; 8 dpi, p = 0.1369; 12 dpi, p = 0.4061) in the virus-exposed mice, the differences were not statistically significant. Only at 15 dpi (*p = 0.0455), the expression of NF-H was significantly decreased in the virus group compared to the control group (Fig 3C). This result indicated that myelin sheath lesions appeared in the early stage of the disease and the axonal damage appeared later in the disease.

### The ultrastructure alterations of myelin and axons in the sciatic nerves appeared at different time

**Fig 4:**
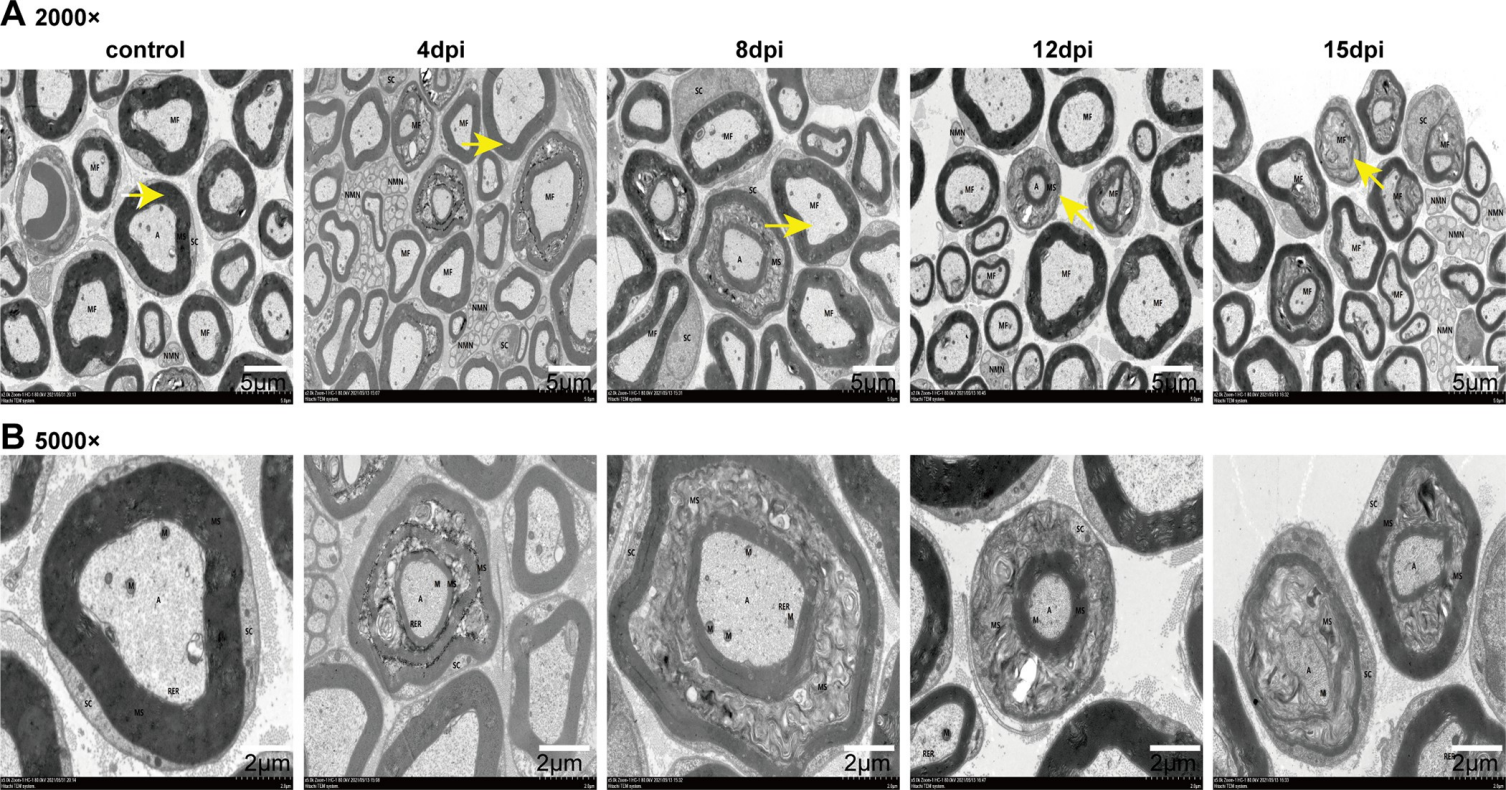
Ultrastructural of sciatic nerve of mice at different time. (A) 2000 × Magnification; (B) 5000 × Magnification. Magnified image of the arrowhead in (A). (MF) Myelinated nerve fiber, (NMN) Nonmyelinated nerve, (MS) myelin sheath, (A) axon, (SC) Schwann cells, (M) mitochondria, (RER) Endoplasmic reticulum structure.

TEM was used to observe the pathological changes in the nerve tissue, which was a more intuitive analysis method. The sciatic nerves exhibited a complete myelin sheath (MS) around the axons in the control group. The myelin sheaths were tight, the structure of the axons (A) was intact, and no atrophy and degeneration were observed. Compared with the control group, the sciatic nerve of mice in the virus group exhibited considerable myelin swelling, looseness, and even dysmyelination beginning at 4 dpi. The nerves also exhibited Schwann cell (SC) and mitochondrial (M) edema, and reduced numbers of organelles. Unmyelinated fibers (NMN) were abundant. From 8 dpi to 12 dpi, the myelin lesions were increased and the numbers of non-myelinated nerve fibers were reduced. However, no axon lesions were observed at this stage. At 15 dpi, dysmyelination was still present, and some axons showed atrophy and degeneration (Fig 4). The number of non-myelinated nerve fibers was significantly higher than before. These results suggested that demyelination occurred early in the mouse sciatic nerve after JEV infection, but minor axonal degeneration only during the later phases.

### Western blot analysis of protein expression in mouse sciatic nerves

**Fig 5:**
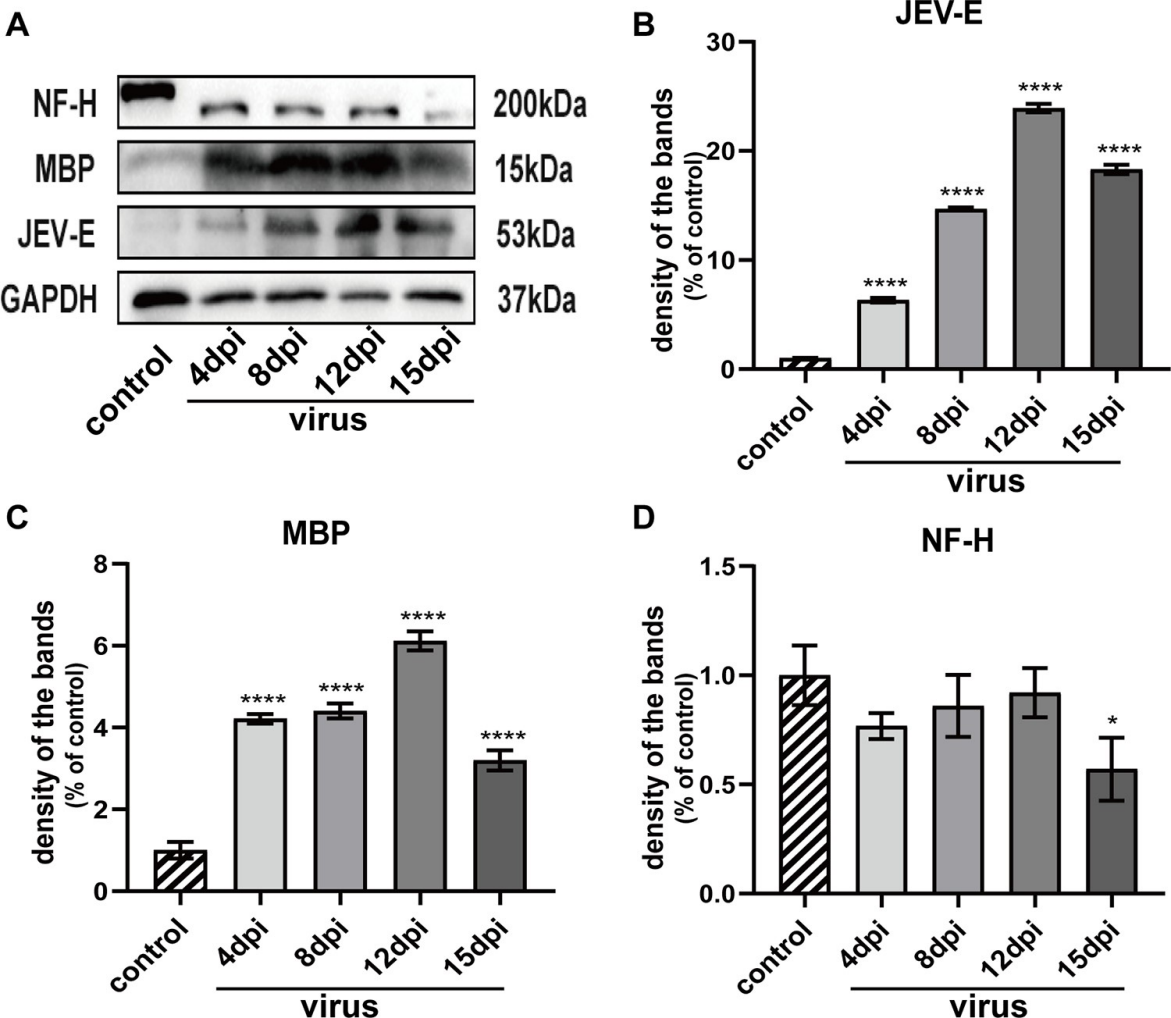
Western blot analysis of related protein expression levels. Statistical analysis by one-way ANOVA. (A) The protein expression was detected by Western blot. Using GAPDH as the reference, the changes of relative expression of different proteins at different time were compared. (B) Relative expression of JEV-E protein at different time; (C) Relative expression of MBP protein at different time; (D) Relative expression of NF-H protein at different time.

The presence and timing of myelin sheath and axonal damage following viral exposure were confirmed by assessing myelin and axon-specific protein expression in sciatic nerves using Western blots. We also detected the expression of the JEV-E protein with the same method (Fig 5A). Each experiment was repeated three times (n = 3), and the data were normalized by the control. Due to the small amount of protein obtained from a single nerve, nerves from five or six mice per group were pooled for each sample. Mice injected with PBS were used as the control. The relative JEV-E protein relative expression was significantly increased at 4 dpi (****p<0.0001) and progressively increased over time (8 dpi and 12 dpi, ****p<0.0001), but was slightly decreased at 15 dpi (****p<0.0001) (Fig 5B). The relative expression of MBP, which indicated fragment formation after myelin disintegration [42], increased from 4 dpi to 15 dpi (****p<0.0001). Numerous myelin fragments were evident in the early stage of the disease, indicating considerable myelin disintegration and loss. These changes were most apparent at 12 dpi and reduced at 15 dpi. This might be due to the large number of myelin fragments engulfed by macrophages at this stage [43] (Fig 5C). A decrease in NF-H expression was first observed at 15 dpi (*p = 0.0111), indicating that mild axonal damage appeared late in the disease course (Fig 5D).

**Fig 6:**
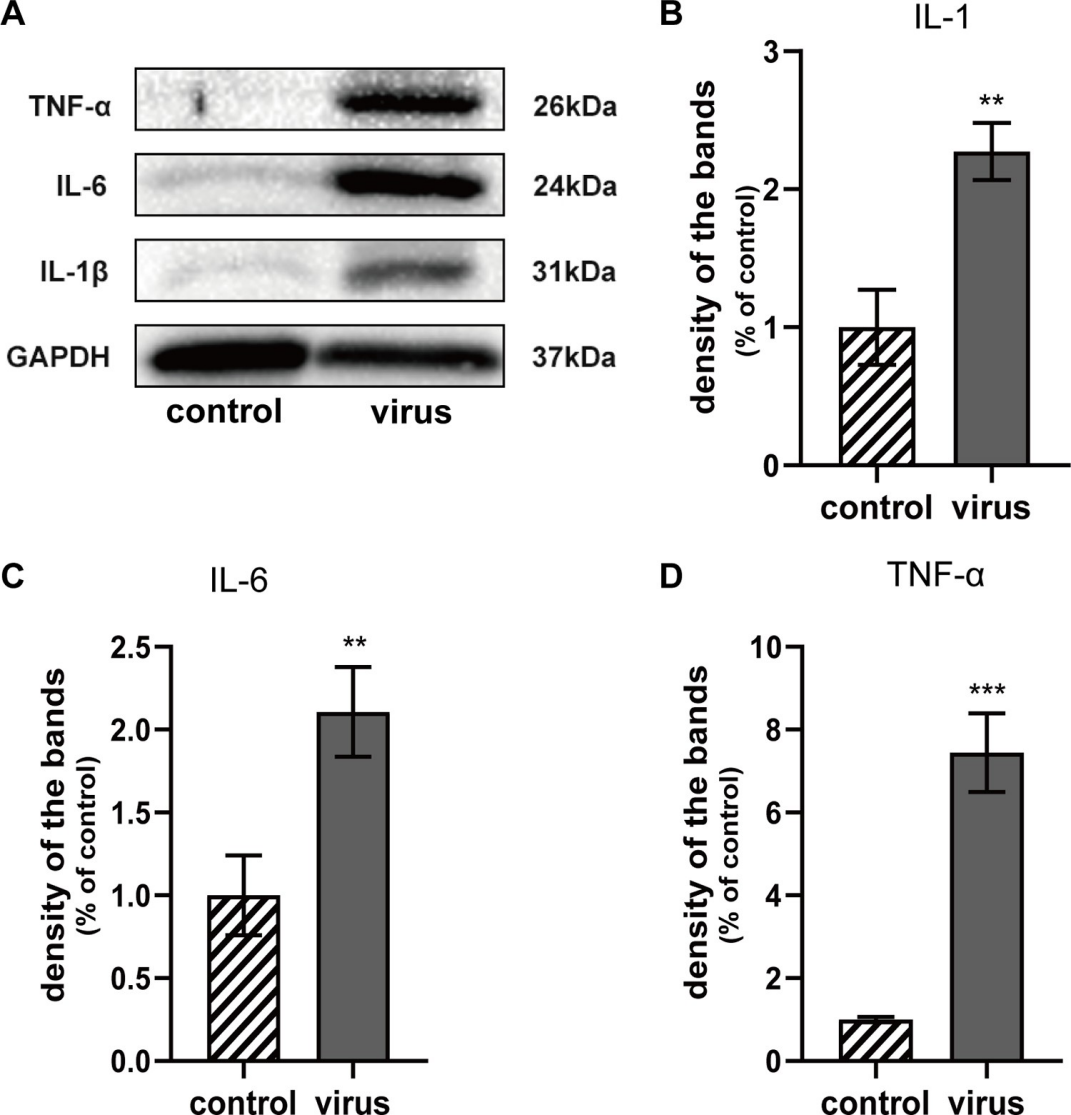
Western blot analysis of Inflammatory cytokines expression levels. (A) Inflammatory cytokines were determined by Western blot; (B) Relative expression of IL-1β between two groups; (C) Relative expression of IL-6 between two groups; (D) Relative expression of TNF-α between two groups.

These results revealed that C57BL/6 mice infected with JEV exhibited primary demyelination with subsequent mild axonal injury. However, we did not know whether the viral infection injured the nerves directly. Thus, in a follow-up experiment, we conducted a proteomic analysis and confirmed that confirmed that the demyelination was caused by an immune response [44]. Several inflammatory cytokines, including IL-1β, IL-6, and TNF-α were identified using Western blots (Fig 6A). The expression of these three cytokines was significantly elevated in the virus group compared with the control group (**p = 0.0029, **p = 0.0061, ***p = 0.0003) (Fig 6B–6D). This result indicated that inflammatory injury occurred in the sciatic nerves.

### Mice exhibited inflammation in the brain after JEV infection

**Fig 7:**
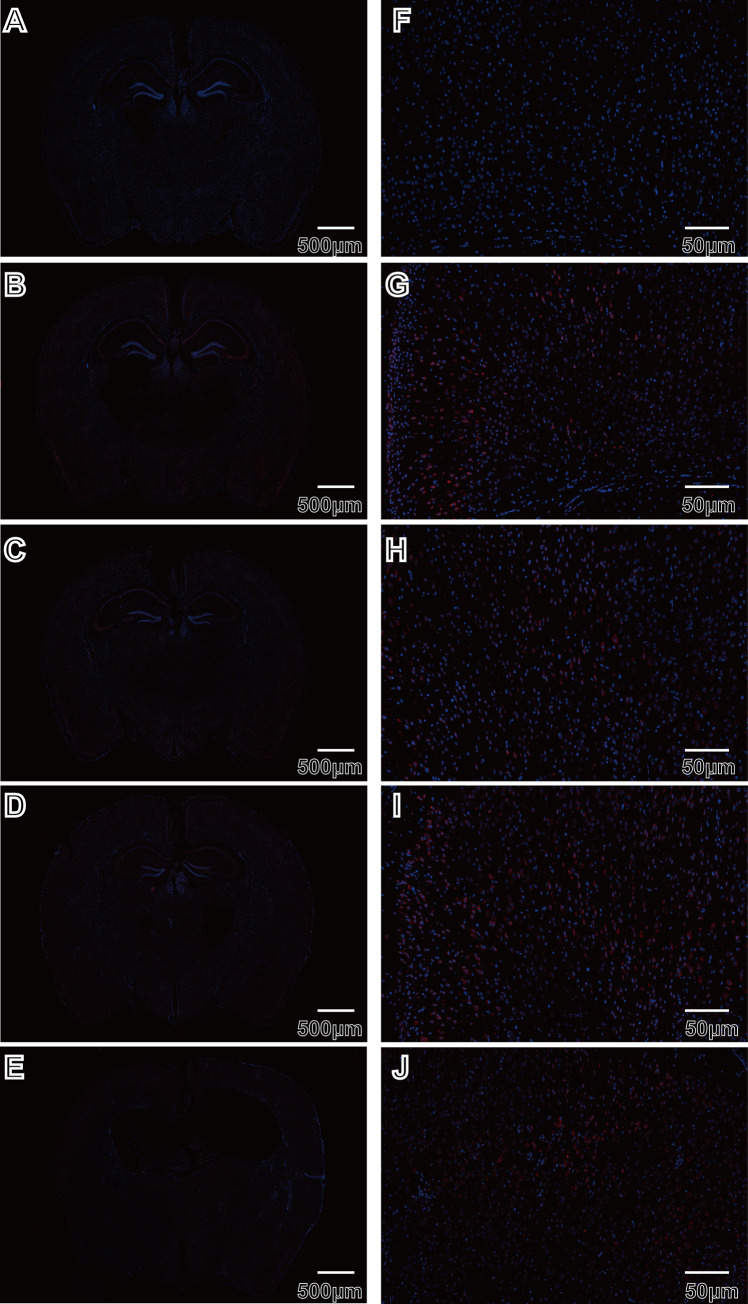
Brain infection were demonstrated by Immunofluorescence. A-J: Immunofluorescence analysis of JEV-E protein of mice brains. A through E (Magnification 3x) and F through J(Magnification 30x). A and F: control group. B through E and G through J: The virus group covered different stages.

JEV tends to infect the brain leading to central nervous system (CNS) lesions, which also can result in limb paralysis. Therefore, we investigated the brains of the mice in this experiment. Three mice with apparent symptoms from the virus-exposed group were evaluated at each time point. Typically, the cerebral cortex is one of the earliest sites infected by JEV [45]. JEV-E protein localization was detected by immunofluorescence. The negative controls (n = 3) did not exhibit any fluorescent staining in their brains (Fig 7A and 7F), but mice developed extensive virus infection in their brains from 4 dpi to 15 dpi (Fig 7B–7E). JEV-E-positive cells were concentrated in the hippocampus and cerebral cortex, with a small number of virus-positive cells present in the white matter. The fluorescein staining for JEV-E did not decrease over time (Fig 7G–7J).

**Fig 8:**
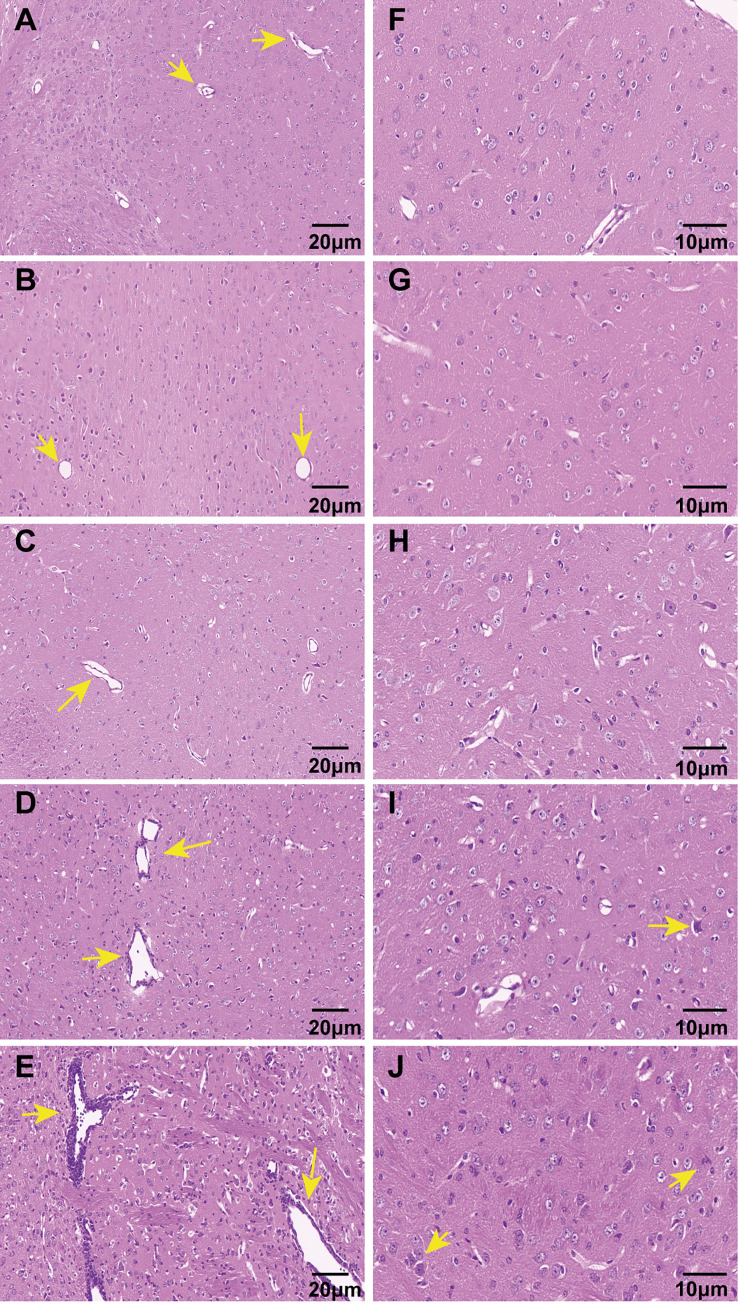
Inflammatory were demonstrated by HE staining of the brains. A through J:HE staining of mice brains. A through E (Magnification 40x) and F through J(Magnification 80x); A and F: control group. B through E and G through J: The virus group covered different stages. No inflammatory cells infiltrated around the vessels (Yellow arrows in A and B); perivascular cuffing (Yellow arrows in C through E); necrosis lesions and glial nodules (Yellow arrows in I and J).

HE staining was used to observe the inflammatory reactions of the brains (n = 3 for all groups). The brain tissues in the control mice displayed normal cellular architecture and no apparent inflammation (Fig 8A and 8F). Although infection in the brains was observed at 4 dpi, no significant signs of inflammation were present (Fig 8B and 8G). At 8 dpi, slight perivascular cuffing was observed (Fig 8C and 8H). This observation was particularly pronounced from 12 dpi to 15 dpi. Infiltration of inflammatory cells, with numerous lymphocytes surrounding blood vessels, was observed. Necrotic lesions and glial nodules also were present (Fig 8D, 8E, 8I and 8J). HE staining revealed that the inflammatory lesions primarily occurred in the thalamus. Although viral-positive cells were mainly found in the cerebral cortex and hippocampus, inflammatory lesions were observed in the cerebral cortex only at 15 dpi. These results indicated that inflammatory response gradually increased in the late stage even though the viral infection of the brain was present at the early stage.

## Discussion

Acute encephalitis syndrome induced by JEV infection is a disease that severely threatens human health in Asia due to the high rates of mortality and disability that are observed [10]. Survivors may develop permanent mental and neurological dysfunction [46,47]. However, the PNI induced by JEV appears to be a new clinical feature or has been overlooked previously.

In this study, after mice were inoculated with JEV, they exhibited a range of symptoms, including weakness, reduced activity, rough hair coat, a hunched back, and body tremors, which were consistent with CNS damage [25,39]. The histological analysis of HE-stained brain sections confirmed these observations. Our experimental findings revealed an inflammatory response in the mouse brains at 8 dpi after JEV inoculation, and the extent of the inflammatory response in the brains gradually increased from 8 dpi to 15 dpi. Limb paralysis was documented in the virus-exposed mice based on the assessment of paralysis and the hanging wire test. These symptoms were most severe at 8–12 dpi, but there was some recovery at 15 dpi. The CNS injury might also exhibit a similar progression, but the time course of the symptom appearance did not support that possibility. Even when the symptoms exhibited by the mice improved, the viral load and the inflammatory response in the brain did not decrease. We performed EMGs to obtain a more accurate determination of whether the limb paralysis was associated with PNI. EMGs are a more accurate method for diagnosing human peripheral neuropathy. Based on the EMG results, we determined that the sciatic nerve conduction velocity in the virus-exposed mice decreased significantly early in the disease, which indicated myelin function, but the latency was not prolonged significantly. Although most mice did not exhibit typical clinical manifestations at this early stage, the decreased nerve conduction velocity demonstrated that demyelination occurred. The amplitude only decreased in the late stage of the disease, indicating that the axon lesions appeared later and might be secondary effects. Myelin sheaths are essential for axons because they provide nutritional support and protection [48]. In addition, infection of motor neurons also can result in axonal degeneration, but we did not investigate this question. However, the EMG results supported our findings. Nerve conduction in both ends of the sciatic nerves was measured using EMG, which reflected the degree of neuropathy in the nerve and allowed the exclusion of motor neuron lesions.

We observed the pathological changes in the sciatic nerves by immunofluorescence and TEM. The observed pathology confirmed that demyelination occurred in the early stage and axonal degeneration occurred only in the later stage of the disease. The MFI of MBP was significantly decreased at the early stage and was minimal at 12 dpi. However, the MFI was slightly increased at 15 dpi. The MFI of NF-H was reduced only at 15 dpi. These results were generally in agreement with the EMG results. The amplitude of sciatic nerve was reduced starting at 12 dpi, which was slightly different from the results obtained with immunofluorescence. This might indicate that dysfunction often occurs before the appearance of pathological changes. Similar results were observed with TEM. We did not find re-myelination of the injured nerve using TEM. The Western blot results suggested the reason for this observation. The MBP expression reflected the formation of myelin fragments after demyelination, and the expression significantly increased in the early stage after virus infection and decreased at 15 dpi. However, the NF-H expression decreased only in the later stage of the disease. Clearance of myelin debris by phagocytes is a critical step in the re-myelination process [49,50], but we were not able to obtain information documenting the phagocytosis of myelin debris by TEM. However, the reduced expression of MBP at 15 dpi suggested that the amount of myelin debris was reduced. The JEV infection became more pronounced from 4 dpi to 12 dpi in nerves that were infected by the virus but decreased slightly at 15 dpi. Currently, we cannot fully determine whether the virus infection directly caused the lesions. However, a subsequent proteomics study indicated that the lesions resulted from tissue damage caused by immune responses [44].

We discovered that JEV could cause peripheral nerve dysfunction in addition to CNS disorders such as encephalitis. The peripheral neuropathy primarily presented as inflammatory demyelination that appeared early in the disease. Axonal lesions took place in the later stage of the disease, which might have resulted from inadequate neurotrophic factors and protection that myelin provided. This result was consistent with the AIDP subtype in GBS [51]. Although axon degeneration occurred, the mice recovered from paralysis in the late stage because the degree of axonal lesions was not severe at this stage. When phagocytes cleared the myelin debris, re-myelination was possible, which was essential for functional recovery after a demyelinating injury. Thus, symptomatic recovery took place in the virus-infected mice.

When flaviviruses invade host cells, the immune response is activated rapidly and triggers inflammation to enable the host defense [52]. This was verified in our subsequent experiment. Using a proteomics analysis, we found that substantial virus-induced immune response-related proteins were up-regulated [44]. The expression of inflammatory cytokines in the sciatic nerve, such as IL-1β, IL-6, and TNF-α, was detected. These inflammatory factors were significantly increased during the JEV infection. It is well known that excessive immune responses can cause tissue damage and harm the host. No mice died in our study, which might have occurred for several reasons: 1) We used C57BL/6 mice, which have a robust immune system [39], allowing them to generate an adequate immune response to clear or reduce the viral load. Numerous studies have utilized A129 or AG129 mice with the interferon receptor knocked out. This protocol improves the viral infection rate but also enhances mortality [53]. 2) We selected subcutaneous viral administration. Many JE studies used intra-cranial injection, which can quickly break through the blood-brain barrier and cause encephalitis [54]. However, we used a subcutaneous route of administration since our study was primarily focused on the peripheral nervous system rather than the CNS. 3) It was necessary to conduct the EMGs in live animals. When mice became very weak, exhibited a significant reduction in body weight, and the VPS was≥4 points, they were euthanized after EMG assessment. Therefore, fatalities due to the disease process were not observed. Approximately one-third of the virus-exposed mice did not exhibit any apparent symptoms or the symptoms were only mild. Because our research focused on PNI, which could develop into chronic disease sequelae or appear at a later stage, it was necessary that the mice exhibit a low mortality rate to identify peripheral neuropathy.

It has been reported that JEV can induce or regulate the immune reactions that result in CNS inflammatory demyelination [55]. Despite having diverse glial cell, the CNS and the peripheral nervous system appeared to display similar types of lesions after JEV infection. This observation provides an important direction for future study.

Our study confirmed that JEV caused not only CNS injury but also PNI that led to limb paralysis. This was the first demonstration that PNI could be caused by JEV infection in mice. JEV can induce immune responses in the host, leading to inflammatory demyelinating peripheral neuropathy. Our research developed a reliable animal model, which is necessary to further explore the mechanism of JEV-induced PNI.

## Supporting information

S1 Fig Behavioral comparison of mice in different virus titer groups. (A) The VPS scores for each group, Kruskal-Wallis H test was used (*p = 0.0233); (B) The VPS scores of the two groups at different time points. Kruskal-Wallis H test was used. (**p = 0.0033).
(TIF)

S2 Fig Body weight mean of two goups at different time. (TIF)

S1 Data Excel spreadsheet containing, in separate sheets, the underlying numerical data for Figure panels 1A, 1B, 1C, 2A, 2B, 2C, 3B, 3C, 5B, 5C, 5D, 6B, 6C, 6D. (XLSX)

S2 Data Excel spreadsheet containing, in separate sheets, the underlying numerical data for supporting Figure panels S1A, S1B, S2. (XLSX)
