# Supplementary figures and images for: A mouse model of peripheral nerve injury induced by Japanese encephalitis virus
Source: PLoS Negl Trop Dis. 2022 Nov 28;16(11):e0010961. doi: 10.1371/journal.pntd.0010961 (PMC9731479; doi:10.1371/journal.pntd.0010961)

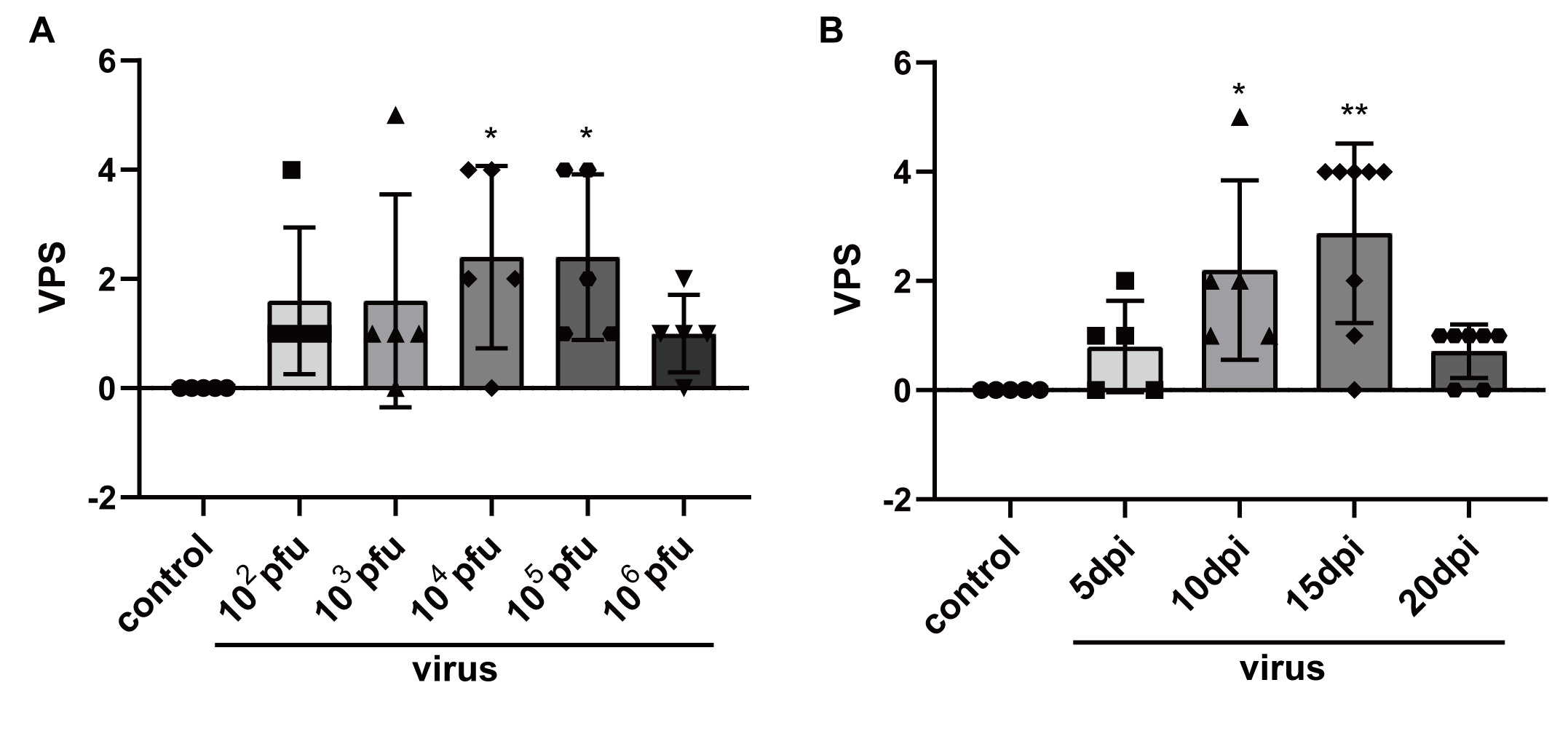

Supplement: S1 Fig — (A) The VPS scores for each group, Kruskal-Wallis H test was used (*p = 0.0233); (B) The VPS scores of the two groups at different time points. Kruskal-Wallis H test was used. (**p = 0.0033). (TIF) [file pntd.0010961.s001.tif]

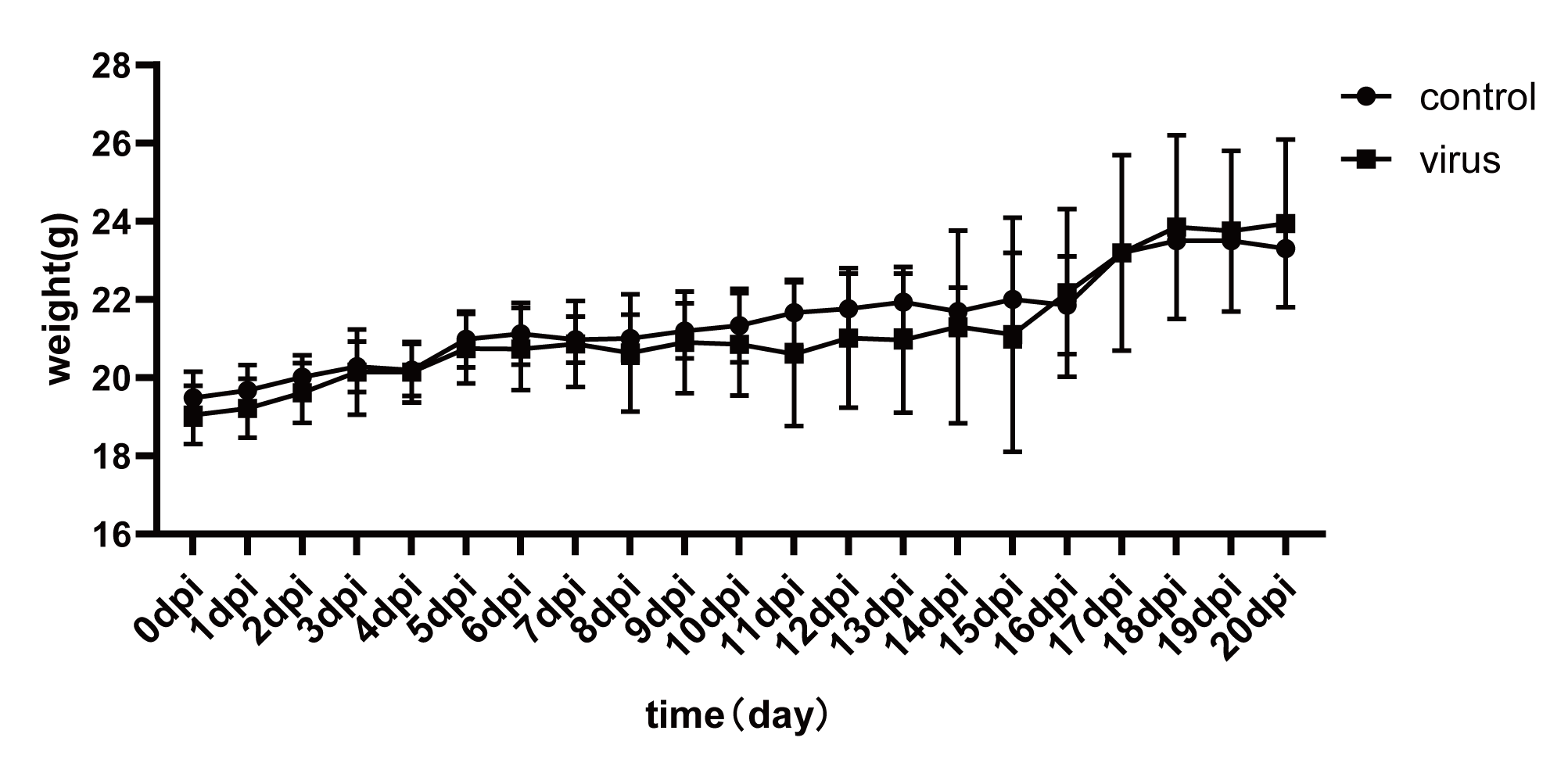

Supplement: S2 Fig — (TIF) [file pntd.0010961.s002.tif]
